# Emotional Intelligence, Empathy, Self-Esteem, and Life Satisfaction in Spanish Adolescents: Regression vs. QCA Models

**DOI:** 10.3389/fpsyg.2020.01629

**Published:** 2020-07-17

**Authors:** Marian Guasp Coll, Diego Navarro-Mateu, María Del Carmen Giménez-Espert, Vicente Javier Prado-Gascó

**Affiliations:** ^1^Faculty of Teaching and Educational Sciences, Catholic University of Valencia, Valencia, Spain; ^2^Department of Nursing, Faculty of Nursing and Chiropody, University of Valencia, Valencia, Spain; ^3^Social Psychology Department, Faculty of Psychology, University of Valencia, Valencia, Spain

**Keywords:** adolescent, emotional intelligence, empathy, life satisfaction, qualitative comparative analysis, regression models, self-esteem

## Abstract

Adolescence is a complex period, in which the individual is subject to profound emotional, physical, and psychological changes. Healthy development during adolescence is crucial for future positive development; self-esteem and life satisfaction are fundamental. The importance of sociodemographic variables (sex and age), empathy, and emotional intelligence (EI) on self-esteem and life satisfaction was studied, comparing complementary methodologies, regression models, and fuzzy-set qualitative comparative analysis (fsQCA) models. This is a cross-sectional design in a convenience sample of 991 adolescents (528 females, 53.3%; aged between 12 and 19 years; M = 14.01, SD = 1.40) from Spanish schools. Data were collected using the Rosenberg Self-Esteem Scale (RSES), the Satisfaction With Life Scale (SWLS), the Basic Empathy Scale (BES), and Trait Meta-Mood Scale (TMMS)24. The results of the regression models suggest that cognitive empathy, emotional clarity, and emotional repair are the main predictor variables for self-esteem. Meanwhile, the results of the fsQCA suggest that being older and low levels of cognitive empathy, emotional clarity, and emotional repair predict higher levels of self-esteem. On the other hand, life satisfaction in regression models is significantly predicted by the emotional clarity and emotional repair dimensions of the TMMS24 and self-esteem. Meanwhile, in the prediction of life satisfaction, the results of the fsQCA suggest that the most important interactions were high emotional clarity, emotional repair, and low self-esteem. As research has already shown, promoting empathy and EI leads to higher levels of self-esteem and life satisfaction.

## Introduction

Adolescence is a complex period in which the individual is subject to profound emotional, physical, and psychological changes (Fonagy et al., 2004; Bucchianeri et al., 2013; Normandin et al., 2015; Powers and Casey, 2015). It is, therefore, a sensitive period in the development of mental disorders (Ensink et al., 2015; Sharp and Wall, 2017). Healthy development during adolescence is therefore crucial not only in avoiding problems in the adolescent himself but also for his future development (Huebner et al., 2013). Within positive or healthy development, there appears to be some agreement on the importance of both self-esteem and well-being in general (Alfaro et al., 2015).

In this respect, adequate self-esteem is particularly important since self-esteem influences the way of perceiving and evaluating oneself. It is defined as confidence in oneself, in one’s values or abilities (McCrae and Costa, 2012), and is therefore considered a protective factor in adolescence since it contributes to preserving one’s biological, psychological, and social well-being (Alvarez Aguirre et al., 2010). High self-esteem is considered vitally important for people’s quality of life since it affects the way they value themselves and their relationship with others (John et al., 2010). Healthy self-esteem is considered a protective factor in life and is essential in adolescence, as it influences human motivation and is associated with a wide range of desirable outcomes (Pyszczynski et al., 2004): general psychological adjustment, positive emotion, social confidence, and prosocial behavior among others (Brown and Ryan, 2003; Leary and MacDonald, 2003; Pepping et al., 2013).

Life satisfaction can be considered to be the cognitive component of subjective well-being and is defined as a conscious cognitive judgment of life in which individuals compare their life circumstances with a self-imposed standard (Diener, 2012; Diener et al., 2013). Life satisfaction is closely related to positive personal, psychological, and social outcomes (Özer et al., 2016). Adolescents with high life satisfaction have a better quality of life (Pavot and Diener, 2008), better levels of physical and psychological health (Friedman and Ryff, 2012; Uchino et al., 2016), positive lifestyle habits (Kim et al., 2014), academic success, increased self-confidence, self-efficacy, mental health, and more supportive relationships (Suldo and Huebner, 2006). Self-esteem and satisfaction with life are therefore related concepts (Judge and Bono, 2001; Frederick et al., 2016; Wichstrom and von Soest, 2016) that appear to affect the individual’s physical and mental health (Sowislo and Orth, 2013). Individuals who feel good about who they are likely make positive evaluations about their life in overall terms (Thomaes et al., 2017). Both concepts seem to be quite stable over time (Anusic and Schimmack, 2016), so intervention to improve them could ensure a healthier present and future development among both children and adults. Life satisfaction has also been consistently positively associated with self-esteem (Proctor et al., 2009).

According to the literature, both concepts seem to be influenced by emotional aspects (Proctor et al., 2009). Within the emotional variables, emotional intelligence (EI) and empathy are particularly important and influential in many aspects of the life of individuals (Kong et al., 2014; Özer et al., 2016). EI is defined as the ability to recognize, express, understand, and regulate one’s own emotions and those of others to improve personal growth and quality of life in social relationships (Mayer et al., 2001) or as a measure of a person’s needs, self-awareness capacities, social awareness, and social skills (Goleman et al., 2002). Previous studies have shown how EI relates positively to different measures of psychological well-being and negatively to affective disorders such as anxiety and depression (Zeidner et al., 2012). Likewise, various studies suggest that EI is negatively related to depression and maladaptive coping styles and positively related to social relationships between peer competition and adaptive coping styles (Palmer et al., 2002; Petrides et al., 2006; Mavroveli et al., 2007). Meanwhile, empathy is considered as a multidimensional construction that implies affective and cognitive responses to another person (Jolliffe and Farrington, 2006; Van der Graaff et al., 2018). Affective empathy includes the right emotional response, while cognitive empathy refers to understanding the other person’s state of mind. Empathy in adolescents has been associated with self-esteem and positive social behaviors (Findlay et al., 2006). Despite the importance of these emotional aspects in the self-esteem and well-being of individuals, the literature is scarce, particularly relating to adolescence, and including both components in the same study. In summary, previous research points to a relationship of emotional variables: EI and empathy with self-esteem and life satisfaction in adolescence. Moreover, life satisfaction has also been consistently positively associated with self-esteem. These relationships suggest the following hypotheses: *H1: Higher empathy and EI will predict higher levels of self-esteem. H2: Higher empathy, EI, and self-esteem will predict higher levels of life satisfaction.*

Along with these aspects, the literature suggests that sociodemographic aspects such as age and sex could influence both self-esteem and satisfaction with life. There are studies that indicate differences in levels of empathy according to the sex variable; girls had higher levels in the affective component while there were no differences in the cognitive component according to sex variable (Lafferty, 2004). In addition, other studies (Goldbeck et al., 2007; Salmela-Aro and Tuominen-Soini, 2010; Moksnes and Espnes, 2013) present gender differences for life satisfaction and self-esteem, indicating that males obtain better results for both life satisfaction and self-esteem than females. Moksnes and Espnes (2013) found that self-esteem plays a positive role in the association with adolescent life satisfaction, and this relationship is equally robust for both genders and across ages (Moksnes and Espnes, 2013). The aforementioned shows gender differences for life satisfaction and self-esteem; this relationship is not affected by age, which suggests the following hypotheses: *H3: Males obtain better results for both life satisfaction and self-esteem than females. H4: Self-esteem and life satisfaction are not influenced by age.*

Despite the importance of sociodemographic and emotional aspects in the health of individuals, understood as a combination of self-esteem and satisfaction with life, we were unable to find any study that considers both emotional and demographic aspects simultaneously. This study focuses not only on sociodemographic variables but also EI and empathy on self-esteem. In addition, it involves all these variables on life satisfaction and incorporates two types of methodology. Most of the studies on adolescents have focused on linear models (Vecchio et al., 2007; Çivitci and Çivitci, 2009; Moksnes and Espnes, 2013; Huang and Su, 2014; Chen et al., 2017), ignoring the interactions and the way in which different paths could lead to the same result, which could be evaluated using qualitative comparative analysis (QCA) models (Blackman et al., 2011). These two methodologies are complementary (Ragin, 2008; Eng and Woodside, 2012). Linear regression models indicate the individual contribution of each variable, while the QCA model is based on the combination of variables, considering equifinality, i.e., the different paths that lead to the same results (Ragin, 2008).

The objective of this study was, therefore, to identify the importance of sociodemographic variables (sex and age), empathy, and EI on the psychological health of adolescents (self-esteem and satisfaction with life), comparing complementary methodologies, regression models, and fuzzy-set qualitative comparative analysis (fsQCA) models.

## Methods

### Participants

A convenience sample of 991 adolescents (528 females, 53.3%, and 463 males, 46.7%) aged between 12 and 19 years (M = 14.01, SD = 1.40) was recruited from Spanish schools from the Valencian Community. The majority were in the first (28.4%) or second (27.4%) year of Compulsory Secondary Education, 19.9% were in the third year, and 19.9% in the fourth year; 2.7% were in the first year of high school and 1.5% in the second year of high school. This study respected the fundamental principles of the Declaration of Helsinki (World Medical Association, 2013), with particular emphasis on the anonymization of the data collected, and confidentiality and non-discrimination of participants. After the schools in the Valencian Community had been selected, they were contacted and the project was outlined to them, and then sessions were organized with the psychologist who wanted to participate. The assessment was performed after receiving authorization from both parents of the underage students and the older students themselves. In order to ensure anonymity, the schools that participated voluntarily were given the total number of questionnaires requested according to the number of students that voluntarily agreed to participate, and sealed boxes where the questionnaire could be deposited thereby guarantee the participating subjects’ anonymity. The evaluation was carried out by the psychologist in the classrooms, collectively and during school hours. The participants completed the self-reported questionnaires, which took around 40 min.

### Measures

– Sociodemographic variables of the adolescents and their age, sex, and school year group were registered.
– Self-esteem was measured using the Rosenberg Self-Esteem Scale (RSES; Rosenberg, 1989). This contains 10 items, rated on a 5-point Likert scale from 1 (*strongly agree*) to 5 (*strongly disagree*). The items are divided into two factors: positive self-esteem and negative self-esteem. Sample items from this scale are “I feel that I have a number of good qualities” (positive) “At times, I think I am no good at all” (negative). Higher scores indicate higher self-esteem. The scale showed adequate psychometric properties in the original version (α = 0.89) and previous studies [α = 0.87, Huang et al. (2019), and α = 0.89, Schwinn et al. (2019)], which is also observed in this study (α = 0.81).
– Life satisfaction was assessed using the Satisfaction With Life Scale (SWLS; Diener et al., 1985). It contains five items and a 7-point Likert scale format, from 1 (*strongly disagree*) to 7 (*strongly agree*). A sample item from this scale is “In most ways, my life is close to my ideal.” Higher values indicate greater satisfaction. It had adequate psychometric properties in the original version (α = 0.87) and previous studies [α = 0.71, Bendayan et al. (2013), and α = 0.80, Miller et al. (2019)], which is also observed in this study (α = 0.78).
– *Empathy* was assessed using the Basic Empathy Scale (BES; Jolliffe and Farrington, 2006), which was translated and adapted into Spanish for adolescents (Villadangos et al., 2016). It is composed of 20 items and uses a 5-point Likert scale answer format, ranging from 1 (*strongly disagree*) to 5 (*strongly agree*). The items are divided into two subcategories: Cognitive Empathy, or the ability to rationally understand the emotions of others, and Emotional Empathy, or the ability to have an emotional response similar to that of others. Sample items from this scale are “I get caught up in other people’s feelings easily” (affective) and “I can often understand how people are feeling even before they tell me” (cognitive). A high score indicates a high level of empathy. The instrument has shown adequate internal consistency in the original version (α = 0.79 cognitive empathy; α = 0.85 affective empathy) and previous studies (α = 0.96 cognitive empathy; α = 0.92 affective empathy) (Villadangos et al., 2016). In the present study, internal consistency was a Cronbach alpha of 0.68 for both dimensions of the scale.
– *EI* was evaluated from the perspective of emotional processing using the Trait Meta-Mood Scale (TMMS)24. It is a reduced version of the TMMS48 (Fernandez-Berrocal et al., 2004) and assesses each person’s understanding of his or her emotional states (Inglés et al., 2017) using 24 items with a 5-point Likert scale with anchors of 1 (*do not agree at all*) and 5 (*agree completely*). They are organized into three factors of eight items each: (i) Attention, or the ability to realize one’s own feelings and those of others; (ii) Clarity, or the ability to perceive emotions as well as the cause of emotions; and (iii) Repair, or the ability to regulate one’s own and others’ emotions. Examples of these items are “I pay a lot of attention to my feelings” (attention), “I am usually very clear about my feelings” (clarity), and “Although I am sometimes sad, I have a mostly optimistic outlook” (repair). A high score indicates a high level of EI. All three scales have shown adequate psychometric properties in the original version (all Cronbach alphas were above 0.85) and previous studies. Specifically, emotional attention presented a Cronbach’s α ≥ 0.91. Emotional clarity showed a Cronbach’s α ≥ 0.84. Finally, for emotional repair, Cronbach’s α ≥ 0.86 was observed (Gomez-Baya et al., 2016). Another study showed satisfactory internal consistency α = 0.85 for emotional attention, α = 0.81 for emotional clarity, and α = 0.82 for emotional repair (Inglés et al., 2017). In this study, reliability was α = 0.85 for attention, α = 0.83 for emotional clarity, and α = 0.78 for emotional repair.

### Statistical Analyses

Statistical analysis was carried out using hierarchical regression models and fsQCA models. In the case of hierarchical regression models, three steps were considered for the prediction of self-esteem: sociodemographic variables (sex and age) (step 1), the two dimensions of the BES questionnaire (step 2), and the three dimensions of the TMMS24 (step 3). For the SWLS prediction, and as suggested by the literature, one more step was added to the overall self-esteem using the RSES (step 4). For the calculation of fsQCA prior to the calculation of the necessary and sufficiency analyses, the missing data were eliminated, and the variables were recalibrated with values between 0 (without having the characteristic, completely out of the set, low values) and 1 (having the characteristic, completely in the set, high values). For sex, 0 = man and 1 = woman were assigned.

QCA models identify the percentage of variance explained, or cases where the model is applicable, coverage, as well as indicators of goodness of fit and consistency (Ragin, 2008; Eng and Woodside, 2012). A condition is considered necessary in QCA models when it must always be present for a given result, and its consistency is ≥0.90 (Ragin, 2008). A condition is considered sufficient when the consistency is around or above 0.75 (Eng and Woodside, 2012); it does not always have to be present for a given result and implies a combination of conditions that can lead to a particular result. The values of each variable were subsequently recalibrated with the fsQCA 2.5 software (Claude and Christopher, 2014). To perform this calibration, the three thresholds suggested by the literature (Woodside, 2013) are used: the first (0) considers that observation with this value is totally outside the set (under agreement/value, low level) of the 10% percentile; the second (0.5) considers a midpoint, neither inside nor outside the set (an intermediate level of agreement/value, intermediate level) 50%; and the last value (1) considers that the observation is totally inside the set (high level of agreement/value, high level) 90% percentile (Ragin, 2008; Giménez-Espert and Prado-Gascó, 2018). Necessary and sufficient condition tests were used to evaluate the effect of sociodemographic variables (sex and age), empathy, and EI on the psychological health of individuals (self-esteem and satisfaction with life). The identification of sufficient fsQCA conditions is done through an algorithm that calculates the truth table and then generates three possible solutions: complex, parsimonious, and intermediate (Eng and Woodside, 2012). The intermediate solution is the one recommended in the literature (Ragin, 2008), and it is shown here.

QCA models have advantages. They analyze the logical relationships between associate conditions and an outcome, providing more detailed results (Ragin, 2008; Eng and Woodside, 2012). They do not establish linear relationships. They allow to identify combinations of multiple causes. This means that the conditions that lead adolescents to have a high tolerance for diversity will not necessarily be the same (in the opposite direction) as those that lead to a low tolerance for diversity.

The IBM SPSS Statistics version 24.0 software package (IBM Corp, 2016) was used to calculate the hierarchical regression model (HRM) and calibration values, and the fsQCA 2.5 software package (Claude and Christopher, 2014) was used to calculate fsQCA models.

## Results

### Descriptive Statistics and Calibration Values

The main descriptors and calibration values for the variables studied in fsQCA are shown in Table 1. The different calibration values were calculated by multiplying the items in each dimension of the instruments in order to maximize the variance (Ragin, 2008).

**TABLE 1:** Main descriptions and calibration values.

		**SWLS**	**RSES**	**BES**		**TMMS24**		
	**Age**			**CE**	**EE**	**EA**	**EC**	**ER**
M	14.05	947.56	64,216.95	360,926.71	2,068,338.13	67,646.21	46,816.65	62,161.83
SD	1.41	993.13	476,602.33	442,076.87	4,314,398.33	165,099.19	81,638.78	120,205.57
Min	12	1	1	9	3	1	1	1
Max	19	12,900	9,765,625	360,926.71	39,062,500	4,296,875	619,200	2,484,375
**Calibration values**								
P10	12	40	4	19,200	10,368	450	288	360
P50	14	625	400	187,500	468,750	20,480	13,824	18,432
P90	16	2,500	51,712	1,000,000	6,000,000	200,000	128,000	187,500

*M, mean; SD, standard deviation; Min, minimum; Max, maximum; P10, 10th percentile; P50, 50th percentile; P90, 90th percentile; SWLS, satisfaction with life; RSES, Rosenberg Self-Esteem Scale; BES, Basic Empathy Scale; TMMS24, Trait Emotional Meta-Mood Scale; CE, cognitive empathy; EE, emotional empathy; EA, emotional attention; EC, emotional clarity; ER, emotional reparation.*

### Hierarchical Regression Models

A predictive analysis of the effect of both sociodemographic variables, empathy, and EI on self-esteem and sociodemographic variables, empathy, EI, and self-esteem on the life satisfaction of adolescents was then carried out using hierarchical regression according to the objectives of the study. On the effect of sociodemographic variables, empathy, and EI on self-esteem, the criterion variable was the self-esteem, and the predictor variables were sociodemographic variables (sex and age) and the dimensions of the BES and TMMS24. Three differential steps were established: the sociodemographic variables (sex and age) were included in the first step, the two dimensions of the BES questionnaire were included in the second step, and the three dimensions of TMMS24 were included in the last step.

When evaluating the effect of sociodemographic variables, empathy, EI, and self-esteem on the life satisfaction of adolescents, the criterion variable was the life satisfaction of adolescents and the predictor variables were sociodemographic variables (sex and age), dimensions of the BES, TMMS24, and global self-esteem according to RSES. Four differential steps were established. Sociodemographic variables (sex and age) were included in the first step, the two dimensions of the BES questionnaire were included in the second step, the three dimensions of TMMS24 were included in the third step, and global self-esteem was included in the last step.

When evaluating the effect of sociodemographic variables (sex and age), empathy and EI accounted for 25% of self-esteem (*R*^2^_*adjusted*_ = 0.25, *p* ≤ 0.001), and the effect of sociodemographic variables (sex and age), empathy, EI, and self-esteem explained 36% of life satisfaction (*R*^2^_*adjusted*_ = 0.36, *p* ≤ 0.001) (Table 2).

**TABLE 2:** Hierarchical regressions for the dimensions of BES, TMMS24, RSES, and SWLS.

**Variable**	**RSES**		**SWLS**	
**Predictors**	Δ*R*^2^	**β**	Δ*R*^2^	**β**
Step 1	0.01**		0.00	
Sex		0.02		–0.05
Age		0.10**		–0.04
Step 2	0.03***		0.04***	
Sex		0.01		–0.04
Age		0.13***		–0.10
Emotional empathy		–0.01		0.10**
Cognitive empathy		−0.18***		0.14***
Step 3	0.21***		0.19***	
Sex		0.01*		–0.04
Age		0.06		–0.04
Emotional empathy		0.02		0.06
Cognitive empathy		−0.09**		0.05
Emotional attention		0.07*		–0.05
Emotional clarity		−0.25***		0.24***
Emotional repair		−0.34***		0.31***
Step 4		−	0.14***	
Sex		−		–0.03
Age		−		–0.01
Emotional empathy		−		0.07
Cognitive empathy		−		0.02
Emotional attention		−		–0.02
Emotional clarity		−		0.14***
Emotional repair		−		0.17***
RSES		−		−0.43***
Total *R*^2^_*adjusted*_	0.25***	−	0.36***	

*p ≤ 0.05; **p ≤ 0.01; ***p ≤ 0.001; –, do not calculate according to the theoretical model. SWLS, satisfaction with life; RSES, Rosenberg Self-Esteem Scale; BES, Basic Empathy Scale; TMMS24, Trait Emotional Meta-Mood Scale.

According to the self-esteem that can be found in Table 2, in the first step, the sociodemographic variables significantly increased the variance by 1% (Δ*R*^2^ = 0.01, *p* ≤ 0.01) including the dimensions of the BES scale, accounting for 3% of the variance (Δ*R*^2^ = 0.03, *p* ≤ 0.001), and accounting for 21% of the variance (Δ*R*^2^ = 0.21, *p* ≤ 0.001) with the addition of the TMMS24 variables. In this last step, sex (β = 0.01, *p* ≤ 0.05) and emotional attention (β = 0.07, *p* ≤ 0.05) showed statistically significant and positive beta coefficients for self-esteem. On the other hand, cognitive empathy (β = −0.09, *p* ≤ 0.01), emotional clarity (β = −0.25, *p* ≤ 0.001), and emotional repair (β = −0.34, *p* ≤ 0.001) showed statistically significant and negative beta coefficients for self-esteem.

For life satisfaction shown in Table 2, in the first step, sociodemographic variables did not increase variance, including the dimensions of the BES scale, and accounted for 4% of the variance (Δ*R*^2^ = 0.04, *p* ≤ 0.001), including the TMMS24 variables, which explained 19% of the variance (Δ*R*^2^ = 0.19 *p* ≤ 0.001) and explaining 14% of the variance (Δ*R*^2^ = 0.14, *p* ≤ 0.001) with the addition of self-esteem. In this last step, emotional clarity (β = 0.24, *p* ≤ 0.001) and emotional repair (β = 0.31, *p* ≤ 0.001) showed statistically significant and positive beta coefficients for life satisfaction. On the other hand, self-esteem (β = -0.43, *p* ≤ 0.001) showed statistically significant and negative beta coefficients for life satisfaction among adolescents.

### Fuzzy-Set Qualitative Comparative Analysis

#### Necessary Conditions

Table 3 shows the test of necessary conditions, none of the associate conditions can be considered necessary for high or low levels of self-esteem and life satisfaction since the consistency is below 0.90 (Ragin, 2008).

**TABLE 3:** Necessity analysis for RSES and SWLS.

	**RSES**		**∼RSES**		**SWLS**		**∼SWLS**	
	**Cons**	**Cov**	**Cons**	**Cov**	**Cons**	**Cov**	**Cons**	**Cov**
Girl	0.57	0.41	0.51	0.59	0.53	0.48	0.54	0.54
Boy	0.43	0.36	0.49	0.64	0.47	0.47	0.46	0.53
Age	0.65	0.51	0.57	0.71	0.59	0.55	0.62	0.68
∼Age	0.63	0.49	0.61	0.73	0.66	0.60	0.59	0.62
Cognitive empathy	0.54	0.47	0.56	0.76	0.62	0.63	0.52	0.61
∼Cognitive empathy	0.72	0.52	0.61	0.68	0.62	0.53	0.69	0.68
Emotional empathy	0.57	0.52	0.51	0.73	0.59	0.63	0.50	0.63
∼Emotional empathy	0.70	0.50	0.66	0.71	0.66	0.53	0.71	0.67
Emotional attention	0.56	0.51	0.52	0.73	0.58	0.61	0.51	0.63
∼Emotional attention	0.70	0.48	0.65	0.70	0.65	0.53	0.69	0.65
Emotional clarity	0.46	0.41	0.59	0.83	0.66	0.70	0.45	0.55
∼Emotional clarity	0.81	0.56	0.58	0.62	0.57	0.47	0.76	0.72
Emotional repair	0.46	0.41	0.59	0.83	0.67	0.71	0.44	0.55
∼Emotional repair	0.81	0.56	0.58	0.62	0.57	0.47	0.76	0.73
RSES	–	–	–	–	0.36	0.43	0.61	0.85
∼RSES	–	–	–	–	0.87	0.66	0.58	0.51

*RSES, Rosenberg Self-Esteem Scale; SWLS, Satisfaction with the life; Cons, consistency; Cov, coverage; ∼, absence of condition (low levels). Condition needed: consistency ≥0.90;− do not calculate according to the theoretical model.*

#### Sufficient Conditions

Table 4 shows the models resulting from the sufficiency analyses, with consistency around or above 0.75 being a sufficient condition (Eng and Woodside, 2012).

**TABLE 4:** Summary of the three main sufficient conditions for the intermediate solution for RSES and SWLS.

**Frequency cutoff: 1**	**RSES**			**∼RSES**			**SWLS**			**∼SWLS**		
	**Consistency cutoff: 0.80**			**Consistency cutoff: 0.88**			**Consistency cutoff: 0.88**			**Consistency cutoff: 0.90**		
	**1**	**2**	**3**	**1**	**2**	**3**	**1**	**2**	**3**	**1**	**2**	**3**
Girl												
Older	⚫	⚫	⚫		◯	◯						
CE	◯											
EE		⚫	⚫			◯		⚫				
EA	⚫	⚫	⚫		◯				⚫		⚫	
EC	◯	◯	◯	⚫	⚫		⚫		⚫	⚫		◯
ER	◯	◯	◯	⚫		⚫	⚫	⚫	⚫			◯
RSES							◯	◯		⚫	⚫	⚫
Raw coverage	0.27	0.25	0.23	0.42	0.29	0.28	0.48	0.41	0.38	0.52	0.45	0.41
Unique coverage	0.04	0.03	0.01	0.03	0.02	0.00	0.04	0.02	0.01	0.01	0.01	0.00
Consistency	0.81	0.81	0.80	0.89	0.89	0.90	0.84	0.86	0.85	0.89	0.89	0.92
**Overall solution consistency**			**0.73**			**0.83**			**0.77**			**0.86**
**Overall solution coverage**			**0.41**			**0.68**			**0.70**			**0.66**

⚫, *presence of condition;* ◯, *absence of condition (low levels); ∼, absence of condition (low levels); CE, cognitive empathy; EE, emotional empathy; EA, emotional attention; EC, emotional clarity; ER, emotional repair; RSES, Rosenberg Self-Esteem Scale; SWLS, Satisfaction With The Life. Expected address vector for RSES: 1,0,1,1,0,1,1 (0: absent; 1: present) using format (Fiss, 2011). Expected address vector for ∼RSES: 1,1,0,0,1,0,0 (0: absent; 1: present). Expected address vector for SWLS: 0,1,1,1,0,1,1,1 (0: absent; 1: present) using format (Fiss, 2011). Expected address vector for ∼SWLS: 1,1,0,0,1,0,0,0 (0: absent; 1: present). Terms/values in bold, type information on the model as a whole considering all the conditions, not only the most important.*

In the prediction of self-esteem of the adolescents, nine paths were observed that explained 41% of the cases with high levels of self-esteem (Overall Consistency = 0.73; Overall Coverage = 0.41). The most relevant path or combination for predicting high levels of self-esteem (explaining 21% of cases) was the result of the interaction of being older, with high attention to emotions, low cognitive empathy, and low emotional clarity and emotional repair (Raw Coverage = 0.27; Consistency = 0.81). However, in the prediction of low levels of self-esteem in the adolescents, 17 paths were observed that explained 68% of the cases (Overall Consistency = 0.83; Overall Coverage = 0.68). Meanwhile, the most relevant path or combination for predicting low levels of self-esteem (explaining 29% of cases) was the result of the interaction of high emotional clarity, being younger, and low emotional attention (Raw Coverage = 0.29; Consistency = 0.89).

Furthermore, in the prediction of high levels of life satisfaction for the adolescents, 16 paths were examined that explained 70% of the cases with high levels of life satisfaction (Overall Consistency = 0.77; Overall Coverage = 0.70). The most relevant path or combination for predicting high levels of life satisfaction (accounting for 48% of cases) was the result of the interaction of high emotional clarity and emotional repair and low self-esteem (Raw Coverage = 0.48; Consistency = 0.84). Finally, in the prediction of low levels of life satisfaction in the adolescents, 11 paths were observed that explained 66% of the cases (Overall Consistency = 0.86; Overall Coverage = 0.66). Meanwhile, the most relevant path or combination for predicting low levels of life satisfaction (explaining 52% of cases) was the result of the interaction of high emotional clarity and self-esteem (Raw Coverage = 0.52; Consistency = 0.89).

## Discussion

This study extends the research on self-esteem and life satisfaction in adolescence by exploring the importance of sociodemographic variables, empathy, and EI on self-esteem, as well as sociodemographic variables, empathy, EI, and self-esteem on life satisfaction. It compares complementary methodologies, regression models, and fsQCA models.

In general, based on the obtained results, hypothesis one (higher empathy and EI will predict higher levels of self- esteem) can be accepted in the regression model and partially in the fsQCA model. The regression models suggest that sex, cognitive empathy on the BES, and the dimensions of the TMMS24 (emotional attention, emotional clarity, and emotional repair) are significant predictors of self-esteem, as reported in previous studies supporting the influence of emotional skills (empathy and EI), where it was found to be significantly associated with prosocial behaviors, and more positive beliefs about the self (Oberle et al., 2010). The most predictive variable was EI, accounting for 21% of variance explained. In line with the literature, EI has been associated with positive self-esteem and academic, social, and psychological outcomes in adolescence (Di Fabio and Saklofske, 2014; Perera and DiGiacomo, 2015). The most important interactions in predicting self-esteem in sufficiency analyses in the fsQCA, explaining 21% of the cases, were being older, high emotional attention, low cognitive empathy, and low emotional clarity and emotional repair. These results may show that in later adolescence, the cognitive component of empathy, as well as emotional clarity and regulation, plays a less relevant role. These findings can be considered the first step because our study and others show the weak correlations that are usually reported between demographic variables (sex and age), self-esteem, and subjective well-being (Proctor et al., 2009). It highlights the need for future theories and research.

Regarding hypothesis two, higher empathy, EI, and self- esteem will predict higher levels of life satisfaction and can be accepted in the regression model and partially in the fsQCA model. Life satisfaction in the regression model is significantly predicted by the emotional clarity and emotional repair dimensions of the TMMS24 and self-esteem, as shown in the literature, and various environmental variables, sociodemographic variables, and intrapersonal variables such as self-esteem have been recognized as being associated with adolescents’ life satisfaction (Proctor et al., 2009). The most predictable variable again is EI, which explained 19% of the variance. EI influences adaptation to deal with social problems and interpersonal conflicts. Reducing negative emotions, increasing positive ones, and improving emotional regulation promote positive relationships with others, providing positive self-esteem and satisfaction with life (Zeidner and Olnick-Shemesh, 2010; Friedman and Kern, 2014). Meanwhile, in the prediction of life satisfaction, the most important interactions were high emotional clarity and emotional repair and low self-esteem in the fsQCA. These results show the importance of EI, the ability to understand and regulate emotions, and its impact on life satisfaction (Perera and DiGiacomo, 2015; Di Fabio and Kenny, 2016).

No conditions necessary for predicting self-esteem and life satisfaction were observed in the fsQCA. With regard to the third and fourth hypotheses, sex and age on self-esteem and life satisfaction, our results are less conclusive. In regression models, sociodemographic variables increase the variance 1% or nothing. In fsQCA models, age is a condition in predicting high levels of self-esteem. As in other studies, sociodemographic variables are weak predictors (Huebner et al., 2004, 2005), environmental variables are moderate predictors, and intrapersonal variables such as self-esteem are strong predictors (Boden et al., 2008; Deng et al., 2015, 2016; Chen et al., 2017). In general, the results of both methodologies (regression and fsQCA models) indicate emotional variables, empathy, and EI are strong predictors on self-esteem, and all these variables are strong predictors on life satisfaction. When comparing both methodologies, fsQCA models seem to have a higher predictive value than the HRM, and there are variables such as age that appear in the fsQCA and not in the HRM. This is important from the perspective of intervention since some conditions are susceptible to intervention, such as the improvement of emotional skills, self-esteem, and life satisfaction. Adequate self-esteem is associated with life satisfaction (Brown and Ryan, 2003; Leary and MacDonald, 2003; Pepping et al., 2013). EI and empathy encourage prosocial behaviors (Inglés et al., 2014) and are associated with better social relationships (Batanova and Loukas, 2011; Gázquez et al., 2015; Van der Graaff et al., 2018), better mental health (Gomez-Baya et al., 2016), and life satisfaction (Zeidner et al., 2012). In short, promoting empathy and EI, especially focusing on emotional clarity and emotional regulation, in adolescents leads to higher levels of self-esteem and life satisfaction.

In conclusion, this study was an attempt to explore the relationships between empathy, EI, self-esteem, and life satisfaction in a sample of Valencian adolescents. One of the strengths of this study was the comparison of two types of complementary methodologies in predicting self-esteem and life satisfaction of adolescents; fsQCA models involve a greater number of factors than regression models. However, it is not exempt from limitations that will be considered in future research. The type of sampling, not probabilistic, and the inclusion of subjects from only one geographical area make it difficult to generalize the results. Another limitation of this study is the use of self-report for data collection as it may introduce social desirability biases (Dalton and Ortegren, 2011). Despite the limitations of the study, our results show how EI in general and especially emotional clarity and emotional repair and cognitive empathy predict self-esteem in adolescents. In other words, the ability to perceive emotions as well as the cause of emotions and the ability to regulate one’s own and others’ emotions, as well as the ability to understand the other person’s state of mind, predict adolescent self-esteem. In addition, self-esteem and EI, especially emotional clarity and emotional repair, are closely related to positive personal, psychological, and social outcomes and life satisfaction.

This study has implications for theoretical and empirical research of self-esteem related to life satisfaction by identifying conditions, variables, or combinations, such as emotional skills, that appear to have a positive impact on them. The high levels of self-esteem and life satisfaction are psychological resources that promote healthy adaptation in adolescence. Therefore, these results constitute the first step for intervention programs in adolescents’ emotional skills, which in turn will have a direct and positive impact on their self-esteem and life satisfaction, as well as on positive functioning in adolescence.

## Data Availability Statement

The raw data supporting the conclusions of this article will be made available by the authors, without undue reservation.

## Ethics Statement

The studies involving human participants were reviewed and approved by Comité Ético de Investigación en Humanos Universitat de València. Written informed consent to participate in this study was provided by the participants’ legal guardian/next of kin.

## Author Contributions

MG, DN-M, MG-E, and VP-G made substantial contribution to the concept or design of the work or acquisition, analysis, or interpretation of data; drafted the article or revised it critically for important intellectual content; and participated sufficiently in the work to take public responsibility for appropriate portions of the content. All authors contributed to the article and approved the submitted version.

## Conflict of Interest

The authors declare that the research was conducted in the absence of any commercial or financial relationships that could be construed as a potential conflict of interest.
